# A cognitive–affective pathway from socially prescribed perfectionism to athlete burnout: the roles of fear of failure and competitive state anxiety in Chinese athletes

**DOI:** 10.3389/fpsyg.2026.1853398

**Published:** 2026-07-01

**Authors:** Jinrui Zhang

**Affiliations:** School of Sports Training, Chengdu Sport University, Chengdu, Sichuan, China

**Keywords:** athlete burnout, competitive state anxiety, fear of failure, socially prescribed perfectionism, structural equation modeling

## Abstract

**Purpose:**

This study examined whether socially prescribed perfectionism was associated with athlete burnout through a cognitive–affective indirect-effect pattern in Chinese competitive athletes. Fear of failure was conceptualized as the cognitive threat-appraisal component, and competitive state anxiety as the affective competition-proximal component.

**Methods:**

A cross-sectional online survey was completed by 896 Chinese competitive athletes recruited through provincial and municipal training systems across multiple sports. Participants completed standardized Chinese-language measures of socially prescribed perfectionism, fear of failure, competitive state anxiety, and athlete burnout. Confirmatory factor analysis and structural equation modeling were estimated using maximum likelihood, and indirect effects were tested with bias-corrected bootstrapping (5,000 resamples).

**Results:**

The hypothesized model showed good fit (χ^2^/df = 1.995, CFI = 0.977, TLI = 0.975, SRMR = 0.024, RMSEA = 0.033, 90% CI [0.029, 0.037]). Socially prescribed perfectionism was positively associated with athlete burnout (β = 0.28), fear of failure (β = 0.49), and competitive state anxiety (β = 0.11). Fear of failure was positively associated with competitive state anxiety (β = 0.65), and competitive state anxiety was positively associated with athlete burnout (β = 0.58). The direct path from fear of failure to athlete burnout was not significant (β = 0.08, *p* = 0.170). Bias-corrected bootstrapping showed a significant total indirect effect (β = 0.285, 95% CI [0.233, 0.340]), including significant indirect effects through competitive state anxiety alone (β = 0.061, 95% CI [0.015, 0.113]) and through the serial pathway from fear of failure to competitive state anxiety (β = 0.184, 95% CI [0.140, 0.240]). The indirect effect through fear of failure alone was not significant (β = 0.040, 95% CI [−0.016, 0.100]).

**Conclusion:**

Findings provide evidence consistent with, but not causal proof of, a cognitive–affective indirect-effect pattern linking socially prescribed perfectionism to athlete burnout. Competitive state anxiety appeared to be a more proximal correlate of burnout than fear of failure alone, suggesting that interventions in evaluative sport environments may benefit from addressing both failure-related threat appraisals and competition-related anxiety.

## Introduction

Competitive sport is a highly evaluative context in which training and performance are continuously monitored, compared, and judged (Cresswell and Eklund, 2007b; Simpson et al., 2024). Selection opportunities, playing time, scholarships, and public recognition often depend on outcomes that are visible to coaches, teammates, families, and broader audiences. Within such environments, psychological strain may accumulate alongside physical demands, and this pattern has been linked to athlete burnout (Cresswell and Eklund, 2007a). Athlete burnout is typically conceptualized as a multidimensional syndrome characterized by emotional/physical exhaustion, a reduced sense of accomplishment, and sport devaluation (Cresswell and Eklund, 2006; Raedeke and Smith, 2001). Recent review evidence has reinforced the relevance of stress-based accounts of athlete burnout, showing that stress is consistently associated with burnout indicators across athlete samples (Gustafsson et al., 2017; Lin et al., 2022). Because burnout is associated with poorer wellbeing, reduced engagement, and less stable performance over time (Glandorf et al., 2024; Grzelak and Langner, 2024), identifying psychological correlates of burnout remains an important issue in sport psychology. This issue may be especially relevant in the Chinese competitive sport system, where athletes often develop within highly structured provincial and municipal training programs characterized by frequent evaluation, selection pressure, and strong performance expectations from coaches and sport organizations. In such settings, externally imposed standards may be especially salient, making perfectionism—and particularly socially prescribed perfectionism—a meaningful construct for understanding burnout-related risk.

Perfectionism has long been discussed as an important vulnerability factor in sport, but its dimensions do not appear to operate in the same way (Hill and Curran, 2016). Sport-specific meta-analytic evidence has further indicated that perfectionism is meaningfully related to burnout among elite athletes, supporting the relevance of perfectionistic tendencies for understanding burnout-related risk in competitive sport (Yang et al., 2023). Socially prescribed perfectionism refers to the perception that important others expect flawless performance and that social acceptance depends on meeting strict external standards (Hewitt and Flett, 1991). In sport settings, where mistakes are highly visible and feedback is often immediate and consequential, socially prescribed perfectionism may be especially relevant to how athletes interpret performance demands and evaluate errors (Hill et al., 2010; Minichiello et al., 2024). Although socially prescribed perfectionism has been linked to maladaptive outcomes, the processes through which it is associated with athlete burnout remain insufficiently specified. Existing research has often focused on direct associations between perfectionism and burnout or has examined fear of failure and competitive state anxiety in separate models (Hill and Curran, 2016; Hill et al., 2010; Kwon and Cho, 2020). This leaves a more specific theoretical gap: although direct relationships among perfectionism, anxiety, fear of failure, and burnout have been examined, less is known about whether fear of failure and competitive state anxiety operate together as a sequential cognitive–affective process linking socially prescribed perfectionism to burnout symptoms. Fear of failure reflects the tendency to interpret poor performance as threatening because it may lead to shame, reduced self-worth, or loss of regard from important others (Conroy, 2004; Conroy et al., 2002). A recent scoping review has also highlighted fear of failure as a broad and important construct in sport, exercise, and physical activity, with relevance for motivation, avoidance, affective responses, and achievement-related difficulties (Taylor et al., 2023). Competitive state anxiety, by contrast, refers to competition-related worry and physiological arousal in evaluative situations (Ehrlenspiel et al., 2011), and evidence from competitive-anxiety research suggests that anxiety responses may vary as a function of athlete characteristics and sport-context features (Rocha and Osório, 2018). Within the present model, fear of failure is positioned as the cognitive component because it reflects failure-related threat appraisal, whereas competitive state anxiety is positioned as the affective component because it reflects competition-proximal worry, somatic activation, and confidence-related disturbance. Prior findings also leave open the question of whether fear of failure is directly associated with burnout or whether its role is more indirect through proximal emotional responses. Thus, an unresolved issue is whether fear of failure and competitive state anxiety function as a coherent cognitive–affective chain within the association between socially prescribed perfectionism and athlete burnout.

The present study addressed this issue by testing a hypothesized process model in a sample of Chinese competitive athletes, a context in which externally imposed standards and evaluative pressures may be especially salient. The sample was expected to include athletes from different sports, competitive levels, age groups, and training backgrounds; however, these athletes shared participation in organized competitive sport environments where performance is repeatedly evaluated and compared. Accordingly, the study was designed to examine the overall pattern of associations across a broad competitive-athlete sample, without assuming that the strength of each path would necessarily be identical across all athlete subgroups. Rather than making causal claims, this study examined whether the pattern of associations among socially prescribed perfectionism, fear of failure, competitive state anxiety, and athlete burnout was consistent with a cognitive–affective sequence linking perceived external perfectionistic demands, failure-related threat appraisal, competition-related anxiety, and burnout symptoms. By doing so, the study sought to clarify the potential roles of fear of failure and competitive state anxiety in the association between socially prescribed perfectionism and athlete burnout.

## Literature review and hypotheses

The present hypotheses were organized around a cognitive–affective process account of burnout-related risk in competitive sport. Socially prescribed perfectionism was treated as a relatively distal perception of external evaluative pressure, fear of failure was treated as the cognitive component because it reflects threat appraisals concerning the consequences of poor performance, and competitive state anxiety was treated as the affective component because it reflects competition-proximal worry, somatic activation, and reduced confidence. Athlete burnout, in turn, was conceptualized as a longer-term strain outcome associated with repeated exposure to demanding and evaluative sport experiences. Although athletes may differ in sport type, competitive level, age, and training background, the hypotheses were intended to describe the average association pattern among athletes embedded in organized evaluative sport environments, rather than to assume identical path magnitudes across all subgroups. Accordingly, the proposed model was tested as an association pattern and not as evidence of temporal causation.

### Socially prescribed perfectionism and athlete burnout

Perfectionism in sport is commonly conceptualized as multidimensional, with particular attention to the source of standards and the perceived consequences of not meeting them. Socially prescribed perfectionism refers to the perception that important others demand flawless performance, paired with a belief that social approval is contingent on meeting external standards (Hewitt and Flett, 1991). Sport environments can intensify such perceptions because evaluation is frequent, public, and consequential, making external standards highly salient. Meta-analytic evidence has shown that perfectionism is meaningfully related to burnout, especially when perfectionism involves evaluative concerns, perceived pressure, and fear of not meeting external standards (Hill and Curran, 2016; Yang et al., 2023). This evidence is particularly relevant in competitive sport, where performance errors are often visible and may carry consequences for selection, status, and future opportunities.

Athlete burnout is generally viewed as a chronic maladaptive response to prolonged stress, reflected in emotional/physical exhaustion, reduced sense of accomplishment, and sport devaluation (Raedeke and Smith, 2001). Stress-based accounts of burnout propose that enduring demands, coupled with appraisals of insufficient resources and ineffective coping, gradually produce depletion and disengagement (Gustafsson et al., 2011; Smith, 1986). A systematic review and meta-analysis of athlete stress and burnout further supports the view that stress is a robust correlate of burnout symptoms in athletes (Lin et al., 2022). Within this logic, socially prescribed perfectionism can be understood as a persistent interpersonal stressor: performance becomes tied to external evaluation, mistakes are framed as unacceptable, and the psychological cost of imperfection increases. Empirical work across achievement contexts has linked socially prescribed perfectionism to maladjustment and strain, and sport-focused evidence has also connected perfectionistic pressure to burnout-related outcomes (Hill et al., 2008; Madigan et al., 2017). Thus, when athletes believe that important others expect flawlessness and evaluate them conditionally, they may be more likely to experience the exhaustion, reduced accomplishment, and devaluation that characterize burnout.

H1: Socially prescribed perfectionism is positively associated with athlete burnout.

### Socially prescribed perfectionism and fear of failure

Fear of failure is commonly defined as the tendency to appraise failure as threatening because of anticipated aversive consequences, including shame and embarrassment, devaluation of self-worth, loss of interest from important others, upsetting important others, and uncertainty about the future (Conroy et al., 2002). Such appraisals are closely aligned with socially prescribed perfectionism (Gómez-López et al., 2025). A recent scoping review of fear of failure in sport, exercise, and physical activity indicates that fear of failure is not merely a performance concern, but a broader appraisal pattern linked to motivation, avoidance, affective responses, and achievement difficulties (Taylor et al., 2023). This is important for the present model because socially prescribed perfectionism may create precisely the conditions under which failure becomes interpreted as socially and personally costly.

When external standards are perceived as strict and acceptance as conditional, failure is more likely to be interpreted as evidence of inadequacy rather than as a normal feature of learning and skill development. Conditional regard and evaluative climates can amplify perceived consequences of mistakes, increasing the likelihood of failure-related threat appraisals (Donachie et al., 2025). Theoretical perspectives grounded in transactional stress models emphasize that stable orientations shape appraisal patterns in demanding situations (Lazarus, 1984). Socially prescribed perfectionism may therefore be associated with heightened failure appraisals because performance is interpreted through the lens of external judgment and anticipated interpersonal loss (Triguero Martín et al., 2024). Evidence in achievement and sport contexts has supported associations between socially prescribed perfectionism and failure-related concerns, particularly under conditions of evaluative scrutiny and social comparison (Conroy, 2001; Sagar and Stoeber, 2009). In this sense, fear of failure represents the cognitive bridge through which external perfectionistic pressure becomes internalized as a threat to worth, regard, and future standing.

H2: Socially prescribed perfectionism is positively associated with fear of failure.

### Fear of failure and competitive state anxiety

Competitive state anxiety reflects competition-proximal responses involving cognitive worry and somatic arousal, alongside shifts in confidence (Cox et al., 2003; Martens et al., 1990). Cognitive anxiety is particularly sensitive to threat appraisals about performance consequences, whereas somatic anxiety reflects physiological activation during perceived challenge or threat. Fear of failure is therefore relevant to competitive state anxiety because it increases the perceived stakes of errors and heightens anticipatory concern about negative evaluation. Research on competitive anxiety has shown that anxiety responses are associated with both athlete characteristics and sport-context features, highlighting the importance of evaluative conditions in shaping how athletes experience competition (Rocha and Osório, 2018).

Appraisal-based accounts propose that anxiety intensifies when situations are interpreted as threats to valued goals and self-worth, especially when the anticipated consequences involve social judgment or loss (Lazarus, 1991). Fear of failure embeds precisely such consequences, making competitions more likely to be interpreted as threatening (Cen et al., 2025). Under these conditions, competitions may be experienced less as opportunities to demonstrate competence and more as tests in which mistakes could lead to shame, disappointment, or loss of regard. Empirical studies have shown that athletes with stronger fear of failure report higher competition-related worry and tension, particularly in settings perceived as evaluative or high-stakes (Conroy, 2001; Sagar et al., 2007; Lee and Kang, 2024). Accordingly, fear of failure is expected to be positively associated with competitive state anxiety because threat appraisals provide the cognitive basis for anxiety responses during competition.

H3: Fear of failure is positively associated with competitive state anxiety.

### Competitive state anxiety and athlete burnout

Competitive state anxiety is often studied as a proximal predictor of performance, yet its relevance to burnout is supported by stress accumulation accounts (Gustafsson et al., 2011). Repeated elevations in worry and physiological tension can increase perceived effort costs, disrupt attentional control, impair sleep and recovery, and contribute to emotional exhaustion. Over time, sustained anxiety may reduce perceptions of competence and enjoyment, increasing the likelihood of sport devaluation and reduced accomplishment—central facets of burnout (Gustafsson et al., 2011; Raedeke and Smith, 2001). Consistent with this reasoning, meta-analytic evidence indicates that athlete stress is positively related to burnout, suggesting that repeated psychological strain can accumulate into more chronic burnout-related symptoms (Lin et al., 2022).

From a resource-depletion perspective, persistent anxiety demands ongoing self-regulation to manage worry, arousal, and attentional focus (Wu et al., 2022). When such regulation becomes chronic, exhaustion and motivational erosion become more likely. Competitive state anxiety may therefore be particularly important because it is situated close to actual performance episodes: it captures athletes' immediate worry, bodily tension, and confidence-related disturbance in response to impending competition. Sport research has linked anxiety, maladaptive coping, and stress symptoms to burnout indicators, supporting the plausibility of anxiety as a correlate of burnout risk (Gustafsson et al., 2017; Tang et al., 2022; Yang et al., 2024). Thus, athletes who repeatedly experience high levels of competition-related anxiety may be more vulnerable to the emotional exhaustion and disengagement that define burnout.

H4: Competitive state anxiety is positively associated with athlete burnout.

### Fear of failure and athlete burnout

Fear of failure is not only a precursor to anxiety; it may also be associated directly with burnout (Conroy, 2001; Kang and Gong, 2024). From a cognitive-appraisal perspective, failure appraisals can foster avoidance-oriented goals, rigid self-monitoring, and rumination after errors, all of which increase psychological load across training periods. A persistent tendency to interpret setbacks as personally and socially damaging can undermine perceived progress and meaning, encouraging reduced accomplishment and devaluation (Conroy et al., 2002). The broader fear-of-failure literature in sport further suggests that fear of failure is often connected to maladaptive motivation, avoidance, and negative affective experiences, all of which may contribute to strain when they are repeated over time (Sagar and Stoeber, 2009; Taylor et al., 2023).

At the same time, the role of fear of failure in burnout may be more complex than a simple direct association. Fear of failure is primarily an appraisal process: it concerns how athletes interpret the meaning and consequences of possible failure. Burnout, by contrast, reflects a more chronic strain condition. For failure-related appraisals to become linked with burnout symptoms, they may need to be accompanied by repeated emotional activation, self-regulatory burden, or avoidance-oriented coping. Nevertheless, a direct association remains theoretically plausible because athletes who chronically view failure as shameful, self-defining, or socially costly may experience sport participation as psychologically threatening rather than rewarding. In sport, fear of failure has been associated with maladaptive motivational patterns and negative affect, which are frequently implicated in burnout development (Kang and Gong, 2024).

H5: Fear of failure is positively associated with athlete burnout.

### Socially prescribed perfectionism and competitive state anxiety

Socially prescribed perfectionism may also be associated directly with competitive state anxiety, independent of fear of failure. Perceived external demands can heighten pre-competition worry through concerns about evaluation, selection, and disappointment of important others. Such concerns can manifest as anticipatory cognitive anxiety and somatic tension, particularly when performance is framed as a test of worth. This direct pathway is theoretically important because socially prescribed perfectionism may not only shape how athletes appraise possible failure, but also create a more general sense that competition is an externally judged and high-stakes event.

Prior perfectionism research has consistently linked socially prescribed perfectionism to anxiety symptoms, supporting a direct association with competition-proximal anxiety responses (Hewitt and Flett, 1991; Donachie et al., 2018). In competitive sport, this association may be amplified by the public and consequential nature of evaluation. A review and meta-analysis of competitive anxiety also suggests that athlete characteristics and sport-context factors are associated with anxiety responses, indicating that anxiety is partly shaped by the evaluative demands surrounding competition (Rocha and Osório, 2018). Athletes high in socially prescribed perfectionism may therefore enter competitions with heightened concern about external expectations even before specific failure consequences are appraised. For this reason, socially prescribed perfectionism is expected to be positively associated with competitive state anxiety over and above its association with fear of failure.

H6: Socially prescribed perfectionism is positively associated with competitive state anxiety.

### Mediating mechanisms

An indirect-effects account follows logically from stress appraisal and emotion-generation perspectives (Hill et al., 2008; Madigan et al., 2016). Socially prescribed perfectionism indicates chronic exposure to perceived external pressure; fear of failure represents a cognitively grounded threat appraisal of performance outcomes; competitive state anxiety represents the situational affective response that tends to be activated when perceived threat is salient. Each construct occupies a different conceptual position, moving from relatively stable perceived demands to appraisal to state reaction, and then toward longer-term burnout-related strain (Madigan et al., 2016). This ordering is also consistent with Smith's cognitive-affective model of athletic burnout, in which perceived demands, cognitive appraisal, physiological and emotional responses, and coping processes jointly shape burnout-related outcomes (Smith, 1986).

Two single-indirect pathways are therefore plausible. Fear of failure may account for the association between socially prescribed perfectionism and athlete burnout by increasing perceived threat and sustaining maladaptive motivational and emotional patterns. Competitive state anxiety may also account for this association by reflecting proximal strain that, when repeatedly elevated, is linked to burnout symptoms (Kwon and Cho, 2020). In this respect, the two mediators are not interchangeable: fear of failure captures how athletes interpret the possible meaning of failure, whereas competitive state anxiety captures how athletes affectively respond when evaluative competition becomes imminent or salient. The distinction is important because review evidence suggests that both failure-related appraisals and stress/anxiety-related responses are relevant to athlete maladjustment, but they may operate at different points in the stress process (Lin et al., 2022; Taylor et al., 2023).

H7: Fear of failure accounts for the indirect association between socially prescribed perfectionism and athlete burnout.
H8: Competitive state anxiety accounts for the indirect association between socially prescribed perfectionism and athlete burnout.

A serial indirect pathway integrates both mediators in a coherent cognitive–affective sequence (Kang and Gong, 2024; Madigan et al., 2016). Socially prescribed perfectionism is expected to be associated with stronger failure-related threat appraisals (Sagar and Stoeber, 2009), which may in turn be associated with greater competition-proximal anxiety, culminating in higher burnout symptoms through cumulative strain and motivational erosion. In this model, fear of failure is not simply another negative emotion, but a cognitive appraisal of what failure would mean for the athlete's self-worth, social standing, and future opportunities. Competitive state anxiety is not simply another burnout symptom, but an affective state that may translate threat appraisal into repeated worry, somatic activation, and reduced confidence in competition. A serial model is also consistent with distinctions between antecedent appraisals and proximal emotional states in stress process frameworks (Jamieson et al., 2017). Accordingly, the proposed serial pathway reflects a theoretically ordered movement from perceived external pressure to cognitive threat appraisal, from appraisal to affective competition-related strain, and from this strain to burnout symptoms.

H9: Fear of failure, as a cognitive threat-appraisal component, and competitive state anxiety, as an affective competition-proximal response, jointly account for the sequential indirect association between socially prescribed perfectionism and athlete burnout.

**Figure 1:**
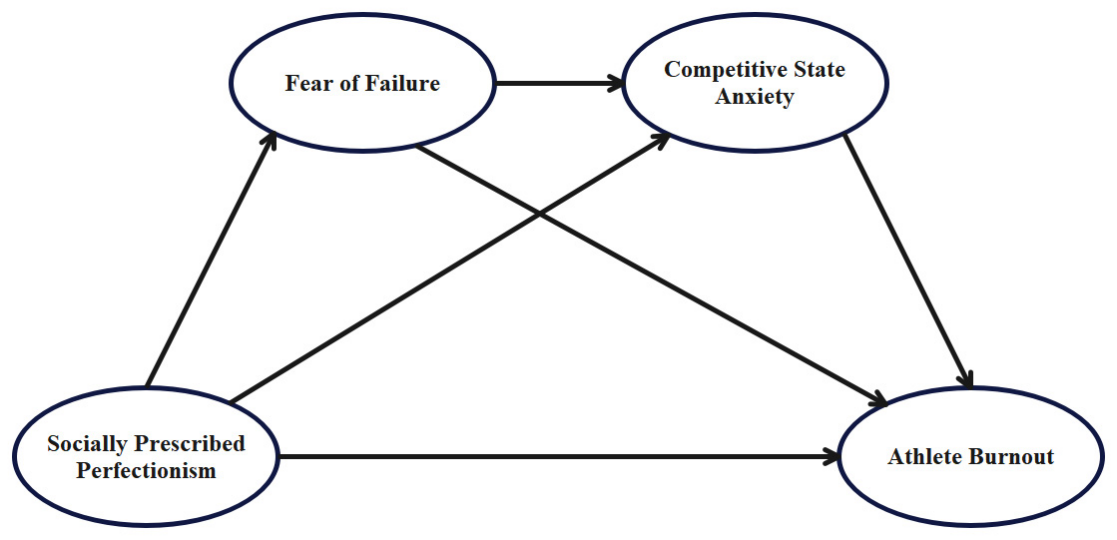
Proposed theoretical model. Fear of failure is conceptualized as the cognitive threat-appraisal component, and competitive state anxiety is conceptualized as the affective competition-proximal response. The proposed cognitive–affective sequence is socially prescribed perfectionism → fear of failure → competitive state anxiety → athlete burnout.

The theoretical model incorporating these direct and indirect pathways is presented in Figure 1.

## Materials and methods

### Participants and sampling

This study used a non-probability purposive and convenience sampling strategy to recruit Chinese competitive athletes from organized sport training settings. Eligible participants were athletes who were currently engaged in regular training and competition within provincial or municipal sport systems. Recruitment was conducted primarily through coaches and team managers from provincial and municipal training systems in Sichuan and Chongqing, with additional participants recruited from other organized competitive sport settings in China.

Before data-quality screening, the intended recruitment endpoint was approximately 1,000 completed questionnaires. This pre-screening target was set to ensure that, after the removal of low-quality responses, the final analytic sample would remain sufficiently large for confirmatory factor analysis, structural equation modeling, and bias-corrected bootstrapping of indirect effects. Sample size requirements in structural equation modeling depend on model complexity, indicator characteristics, factor loadings, missing data patterns, and the magnitude of structural paths rather than on a single universal rule (Wolf et al., 2013). Therefore, the target of approximately 1,000 returned questionnaires was selected to provide a sufficiently large pool for stable latent-variable estimation after screening.

The sampling strategy was also intended to include athletes from different sports, competitive levels, age groups, and training backgrounds. This heterogeneity was consistent with the purpose of the study, which was to examine the overall association pattern among athletes embedded in organized evaluative sport environments. Subgroup differences across sport type, competitive level, gender, and age were not the primary focus of the present study and are addressed as a direction for future research.

### Procedure and data collection

Data were collected between November 1 and November 30, 2025, using the Wenjuan Wang online survey platform. Before recruitment, permission was obtained from the relevant training units and teams. Coaches and team managers distributed the survey link to eligible athletes. Participation was voluntary and anonymous, and no personally identifiable information was collected.

A total of 1,000 questionnaires were returned. Responses were screened to ensure data quality. Questionnaires were excluded if they met one or more of the following criteria: (a) completion time shorter than 300 s, suggesting insufficient engagement; (b) uniform or patterned responding across items, such as identical responses throughout the survey; or (c) failure on embedded attention-check items. Because the Wenjuanwang platform required item completion before submission, the dataset contained no item-level missing values; therefore, missing-data imputation was not required. After data screening, 104 questionnaires were removed. The final analytic sample consisted of 896 athletes. The usable questionnaire proportion among returned questionnaires was 89.6%. Because the total number of athletes who received the forwarded survey link could not be precisely determined, this value was not interpreted as a conventional survey response rate.

The final sample included athletes from more than 10 sport disciplines, including table tennis, badminton, basketball, football, volleyball, swimming, track and field, gymnastics, tennis, weightlifting, martial arts, and shooting. Both individual and team sports were represented, and athletes competed at provincial, national, and international levels. Detailed demographic characteristics are shown in Table 1.

**Table 1:** Demographic characteristics of participants (*N* = 896).

Variable	Category	*N*	%
Gender	Male	512	57.1
	Female	384	42.9
Age	16–18 years	278	31.0
	19–21 years	346	38.6
	22–25 years	192	21.4
	>25 years	80	8.9
Sport type	Individual sports	472	52.7
	Team sports	424	47.3
Competition level	Provincial	401	44.8
	National	371	41.4
	International	124	13.8
Years of training	< 5 years	163	18.2
	5–10 years	384	42.9
	>10 years	349	38.9

### Ethics statement

This study was reviewed and approved by the Ethics Committee of the School of Sports Training, Chengdu Sport University (Approval No. CTYLL2025013). On the first page of the online questionnaire, all participants were provided with an electronic informed consent statement describing the purpose of the study, the voluntary nature of participation, the right to withdraw at any time without penalty, and the confidential and anonymous handling of data. Adult athletes provided electronic informed consent before accessing the questionnaire. For athletes younger than 18 years, electronic informed consent was obtained from a parent or legal guardian, and the adolescent athletes themselves also provided electronic informed consent before participation. Only participants who agreed to the consent statement were able to proceed to the questionnaire. All procedures were conducted in accordance with the ethical standards of the approving committee and the Declaration of Helsinki.

### Measures

Four instruments were used to assess socially prescribed perfectionism, fear of failure, competitive state anxiety, and athlete burnout. All measures were administered in Chinese. For each instrument, prior Chinese-language validation or adaptation evidence was consulted where available (Kou et al., 2010; Cho and Lu, 2005; Li, 2008; Lu et al., 2006; Liu et al., 2022). Because previously available Chinese-language versions differed in item retention, population, response anchors, or regional wording, the present study retained the theoretical content of the original instruments and refined item wording for mainland Chinese competitive athletes. The adaptation procedure applied to all four measures.

The adaptation procedure involved several steps. First, the original English items and available Chinese-language versions were reviewed by bilingual researchers with training in sport psychology. Second, item wording was compared, reconciled, and refined to ensure semantic accuracy, conceptual equivalence, and contextual appropriateness for Chinese competitive athletes. Third, the reconciled Chinese versions were back-translated into English by a bilingual translator who was not involved in the initial wording revision. Fourth, discrepancies between the original and back-translated versions were discussed by the research team and resolved through consensus. Finally, the Chinese wording was reviewed by sport psychology specialists and pilot-checked with competitive athletes to ensure clarity, readability, and cultural appropriateness before formal data collection.

To facilitate comprehension and reduce response burden in a single online survey involving athletes from multiple sports and age groups, all items were presented using a harmonized five-point Likert-type response format, with higher values indicating greater endorsement of the item content. This administration format differed from the original response anchors of some instruments. Therefore, the psychometric adequacy of the adapted measures was evaluated empirically in the current analytic sample through the measurement analyses reported in the Results section. For descriptive analyses, scale scores were calculated after reverse-scoring relevant items where necessary. For the structural equation model, latent-variable indicators were specified as described in the data analysis section.

Socially prescribed perfectionism was assessed using the 15-item Socially Prescribed Perfectionism subscale of the Multidimensional Perfectionism Scale (Hewitt and Flett, 1991). Prior Chinese revision evidence for the Hewitt Multidimensional Perfectionism Scale was consulted to inform wording and cultural adaptation (Kou et al., 2010). The subscale assesses the perception that important others impose excessively high standards and expect flawless performance. A representative item is: “The better I do, the better I am expected to do.” After reverse-scoring the relevant items, higher scores indicated stronger socially prescribed perfectionism.

Fear of failure was measured with the Performance Failure Appraisal Inventory developed by Conroy et al. (2002). Prior Chinese-language adaptation evidence for the PFAI was consulted during wording refinement (Cho and Lu, 2005). The instrument contains 25 items covering five theoretically defined dimensions of anticipated negative consequences of failure: fear of experiencing shame and embarrassment, fear of devaluing one's self-estimate, fear of having an uncertain future, fear of important others losing interest, and fear of upsetting important others. A representative item is: “When I am not succeeding, I am less valuable than when I succeed.” Higher scores indicated stronger failure-related threat appraisals.

Competitive state anxiety was assessed with the Competitive State Anxiety Inventory-2 Revised (CSAI-2R; Cox et al., 2003). Prior Chinese-language validation evidence for the revised CSAI-2R was consulted during adaptation (Li, 2008). The instrument comprises 17 items assessing cognitive anxiety, somatic anxiety, and self-confidence. To preserve the state-referent nature of the construct, the scale was administered with explicit reference to athletes' feelings about their nearest upcoming official competition. Participants were instructed to respond according to how they currently felt when thinking about that competition. A representative somatic-anxiety item is: “My body feels tense.” Because the CSAI-2R includes self-confidence as a positively framed dimension, self-confidence items were reverse-scored before structural modeling so that higher values on all three subdimensions reflected greater competition-related anxiety. In the measurement and structural models, cognitive anxiety, somatic anxiety, and reverse-scored self-confidence were treated as indicators of a broader latent construct representing competitive state anxiety. This operationalization was adopted to capture an overall maladaptive pre-competition affective state in the hypothesized model.

Athlete burnout was measured using the Athlete Burnout Questionnaire (Raedeke and Smith, 2001). Prior Chinese-language ABQ adaptation and validation studies were consulted during wording refinement (Lu et al., 2006; Liu et al., 2022). The instrument contains 15 items assessing three components of burnout: emotional and physical exhaustion, reduced sense of accomplishment, and sport devaluation. A representative item is: “I feel so tired from my training that I have trouble finding energy to do other things.” After reverse-scoring relevant items where necessary, higher scores represented greater burnout symptoms.

### Data analysis

All statistical analyses were conducted using IBM SPSS Statistics 26.0 and AMOS 26.0. SPSS was used for descriptive statistics, data diagnostics, scale scoring, and bivariate correlations. AMOS was used for confirmatory factor analysis (CFA) and structural equation modeling (SEM). Invalid responses had already been removed during the data-screening stage described above.

Descriptive statistics were computed for socially prescribed perfectionism, fear of failure, competitive state anxiety, and athlete burnout. Scale and subscale internal-consistency coefficients were calculated at the appropriate scoring level and are reported with the measurement results in the Results section. Composite reliability (CR) and average variance extracted (AVE) were calculated at the latent-construct level based on the CFA measurement model to assess construct reliability and convergent validity (Fornell and Larcker, 1981). CR values of 0.70 or above, AVE values of 0.50 or above, and standardized factor loadings of 0.60 or above were treated as indicative of acceptable measurement quality.

A joint four-factor CFA measurement model including all four latent constructs was estimated before the structural model. The same measurement specification was then retained in the SEM. Separate CFAs for each instrument were not used as the primary measurement test because the focal analytic question concerned the distinctiveness and structural relations of the four constructs within a single latent-variable model. The measurement model was specified in a theory-driven manner to reflect the conceptual structure of each construct rather than through data-driven item parceling. Socially prescribed perfectionism was modeled using its 15 observed items as indicators because the focal subscale was treated as a relatively unified construct and retaining item-level indicators preserved content specificity. By contrast, fear of failure, competitive state anxiety, and athlete burnout were modeled using their theoretically defined subdimensions as indicators of broader latent variables. Thus, the five fear-of-failure dimensions, the three CSAI-2R subdimensions, and the three athlete-burnout dimensions served as indicators of their respective latent constructs. This mixed-level specification was adopted to preserve theoretical meaning while reducing unnecessary parameter complexity in the structural model.

For competitive state anxiety, special attention was given to the treatment of self-confidence. Because the CSAI-2R conceptualizes self-confidence as a positively framed dimension opposite in direction to anxiety, the self-confidence subscale was reverse-scored before latent-variable modeling. As a result, higher values on cognitive anxiety, somatic anxiety, and reverse-scored self-confidence consistently reflected greater competition-related maladjustment. These three subdimensions were then modeled as indicators of a broader latent construct representing competitive state anxiety in the hypothesized model.

CFA and SEM parameters were estimated using maximum likelihood estimation in AMOS 26.0. Because the items were administered using a five-point Likert-type format and the sample size was large, observed indicators were treated as approximately continuous for model estimation, consistent with common SEM practice for multi-category ordinal indicators under sufficiently large samples (Rhemtulla et al., 2012). Indirect effects were examined using bias-corrected bootstrapping, which does not assume normality of the indirect-effect sampling distribution.

Given that SEM test statistics can be sensitive to nonnormality and model specification, model fit was evaluated using multiple indices (Curran et al., 1996): the chi-square to degrees of freedom ratio (χ^2^/df), Comparative Fit Index (CFI), Tucker–Lewis Index (TLI), Standardized Root Mean Square Residual (SRMR), and Root Mean Square Error of Approximation (RMSEA) with its 90% confidence interval. Following conventional recommendations, χ^2^/df values below 5.00, CFI and TLI values of 0.90 or above for acceptable fit and 0.95 or above for good fit, SRMR values below 0.08, and RMSEA values below 0.08 for acceptable fit and below 0.05 for close fit were used as evaluation criteria (Browne and Cudeck, 1992; Hu and Bentler, 1999; Marsh and Hocevar, 1985; Marsh et al., 2004). To further examine discriminant validity, the hypothesized four-factor model was compared with alternative one-factor, two-factor, and three-factor models. Discriminant validity was also examined using the Fornell–Larcker criterion.

Because all focal variables were assessed through self-report questionnaires, potential common method bias was examined using procedural and statistical diagnostics. Procedurally, the survey was anonymous, participation was voluntary, and standardized instructions were used. Statistically, Harman's single-factor test was first conducted through unrotated exploratory factor analysis to evaluate whether a single factor accounted for the majority of covariance among the measures (Podsakoff and Organ, 1986; Podsakoff et al., 2003). Second, the fit of the hypothesized four-factor measurement model was compared with that of a single-factor CFA model. Third, an unmeasured latent method construct (ULMC) model was estimated as a supplementary CFA-based sensitivity check. In the ULMC model, indicators were specified to load on their theoretical latent factors, while an additional latent common-method factor was included to capture residual covariance shared across indicators. These procedures were treated as diagnostic checks rather than definitive tests.

Pearson correlation analyses were conducted to examine bivariate relationships among the four study constructs. The hypothesized structural model was then tested using SEM. The model included the direct paths specified in the hypotheses as well as the indirect pathways from socially prescribed perfectionism to athlete burnout through fear of failure and competitive state anxiety. Structural-model fit was evaluated using the same criteria described for the measurement model. Indirect effects were examined using bias-corrected bootstrapping with 5,000 resamples. An indirect effect was considered statistically significant when its 95% confidence interval did not include zero (Shrout and Bolger, 2002).

No demographic covariates were included in the primary SEM analyses. Gender, age, sport type, competition level, and years of training were summarized descriptively to characterize the sample, but they were not entered as routine statistical controls because the purpose of the study was to test a theoretically specified process among the focal psychological constructs. In the absence of strong a priori theory indicating that these background characteristics should be treated as common covariates across all structural paths, including multiple controls was considered likely to reduce model parsimony and complicate interpretation without directly serving the central research question. In this study, these variables were therefore treated as sample descriptors rather than default control variables.

## Results

### Descriptive statistics and measurement reliability

Descriptive statistics and measurement-quality indices for the four study constructs are presented in Table 2. The reliability and validity results supported the adequacy of the adapted measures in the current analytic sample. Internal consistency was satisfactory for all constructs. Standardized factor loadings were within acceptable ranges, composite reliability values exceeded the recommended threshold of 0.70, and average variance extracted values exceeded the recommended cutoff of 0.50. These results supported adequate construct reliability and convergent validity for socially prescribed perfectionism, fear of failure, competitive state anxiety, and athlete burnout.

**Table 2:** Descriptive statistics, reliability, and validity indices of study variables (*N* = 896).

Variable	Mean	SD	α	Factor loading	CR	AVE
Socially prescribed perfectionism	2.44	0.91	0.953	0.723–0.844	0.953	0.577
Fear of failure	2.47	0.75	0.852–0.923	0.747–0.765	0.870	0.573
Competitive state anxiety	2.50	0.78	0.856–0.903	0.698–0.793	0.783	0.546
Athlete burnout	2.38	0.70	0.840–0.849	0.727–0.824	0.808	0.585

SD, Standard Deviation; α, Cronbach's Alpha; CR, Composite Reliability; AVE, Average Variance Extracted.

All reliability and validity indices were calculated using the current analytic sample. For fear of failure, competitive state anxiety, and athlete burnout, α values are presented as ranges across their theoretically defined dimensions or subdimensions.

### Common method bias diagnostics

Because all focal variables were assessed through self-report questionnaires, several procedural and statistical steps were used to reduce and diagnose potential common method bias. At the procedural level, the survey was administered anonymously through an online platform, participants were informed that their responses would be used only for research purposes and treated confidentially, participation was voluntary, and respondents were told that there were no right or wrong answers. Standardized instructions, clear item wording, and established measurement instruments were also used to reduce the likelihood that shared method variance would systematically inflate associations among the study variables.

At the statistical level, common method bias was examined using multiple diagnostic procedures. First, Harman's single-factor test was conducted using unrotated exploratory factor analysis. A total of 12 factors with eigenvalues greater than 1 were extracted, accounting for 65.04% of the cumulative variance. The first factor explained 27.89% of the total variance, suggesting that a single factor did not account for the majority of covariance among the measures.

Second, confirmatory factor analyses were used to compare alternative measurement models. As shown in Table 3, the hypothesized four-factor model showed excellent fit, whereas the one-factor model demonstrated poor fit. Third, an unmeasured latent method construct (ULMC) model was estimated as a supplementary sensitivity check. In this model, the substantive indicators were specified to load on their theoretical latent factors, while an additional latent common-method factor was included to capture variance shared across indicators beyond the focal constructs. The ULMC model showed virtually identical fit to the hypothesized four-factor model and did not yield a substantively meaningful improvement in fit. Taken together, the procedural remedies and statistical diagnostics suggest that common method bias was unlikely to fully account for the observed findings, although it cannot be ruled out entirely.

**Table 3:** Comparison of alternative measurement models (*N* = 896).

Model	χ^2^/df	CFI	TLI	SRMR	RMSEA (90% CI)
One-factor	9.639	0.799	0.782	0.100	0.098 (0.095, 0.102)
Two-factor	8.530	0.825	0.810	0.098	0.092 (0.088, 0.095)
Three-factor	6.511	0.873	0.861	0.087	0.078 (0.075, 0.082)
Four-factor	1.995	0.977	0.975	0.024	0.033 (0.029, 0.037)
ULMC-factor	1.994	0.977	0.975	0.024	0.033 (0.029, 0.037)

ULMC, Unmeasured Latent Method Construct; CFI, Comparative Fit Index; TLI, Tucker–Lewis Index; SRMR, Standardized Root Mean Square Residual; RMSEA, Root Mean Square Error of Approximation; CI, Confidence Interval.

### Correlation analysis and discriminant validity

Pearson correlation analysis was conducted to examine the bivariate relationships among the four study constructs. As shown in Table 4, socially prescribed perfectionism, fear of failure, competitive state anxiety, and athlete burnout were all positively correlated with one another, and all correlations were statistically significant in the expected directions.

**Table 4:** Correlations and discriminant validity among study variables (*N* = 896).

Variable	Socially prescribed perfectionism	Fear of failure	Competitive state anxiety	Athlete burnout
Socially prescribed perfectionism	0.760			
Fear of failure	0.433^***^	0.757		
Competitive state anxiety	0.355^***^	0.553^***^	0.739	
Athlete burnout	0.476^***^	0.494^***^	0.558^***^	0.765

Values on the diagonal represent the square roots of the average variance extracted.

^***^p < 0.001.

Discriminant validity was evaluated using the Fornell–Larcker criterion. The square roots of the average variance extracted are presented on the diagonal of Table 4. For each construct, the square root of the average variance extracted exceeded the corresponding inter-construct correlations, supporting adequate discriminant validity among socially prescribed perfectionism, fear of failure, competitive state anxiety, and athlete burnout.

### Structural equation modeling analysis

**Figure 2:**
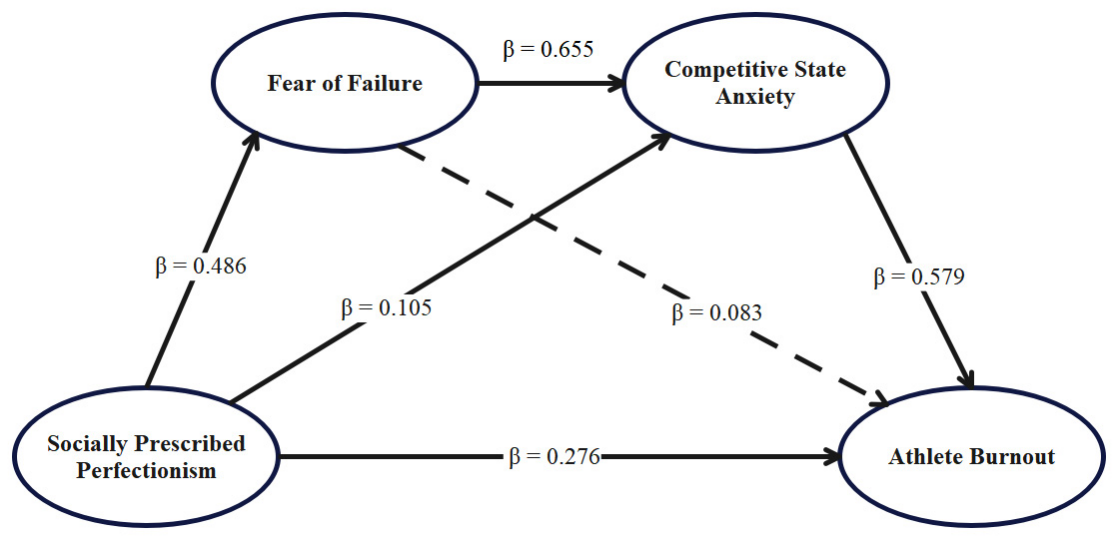
Simplified structural model with standardized path coefficients (*N* = 896). Standardized coefficients are displayed to three decimal places. Solid lines indicate statistically significant paths, and the dashed line indicates a non-significant path. Measurement indicators, residuals, and covariances are omitted to improve readability. The dashed path from fear of failure to athlete burnout was not statistically significant (β = 0.083, *p* = 0.170).

Using maximum likelihood estimation, structural equation modeling was conducted to evaluate the hypothesized structural model. The model showed good fit to the data (χ^2^/df = 1.995, CFI = 0.977, TLI = 0.975, SRMR = 0.024, RMSEA = 0.033, 90% CI [0.029, 0.037]). Standardized path coefficients are presented in Figure 2.

As shown in Figure 2, socially prescribed perfectionism was positively associated with athlete burnout (β = 0.276), fear of failure (β = 0.486), and competitive state anxiety (β = 0.105). Fear of failure was positively associated with competitive state anxiety (β = 0.655), and competitive state anxiety was positively associated with athlete burnout (β = 0.579). The direct path from fear of failure to athlete burnout was not statistically significant (β = 0.083, *p* = 0.170). All remaining structural paths were statistically significant.

It is noteworthy that the fit indices of the structural model were identical to those of the hypothesized four-factor measurement model. This pattern is attributable to the fact that, at the latent-variable level, the structural portion of the model was effectively just-identified. That is, the specified directional paths among the four latent constructs reproduced the same degree of freedom as the freely estimated interfactor associations in the four-factor CFA. Accordingly, the identical fit indices do not indicate a model estimation problem. Instead, overall model fit was primarily determined by the adequacy of the measurement model, whereas interpretation of the structural model rests mainly on the magnitude and statistical significance of the estimated paths.

Compared with the theoretical model presented in Figure 1, the structural results in Figure 2 provided partial support for the proposed model. The paths from socially prescribed perfectionism to fear of failure, competitive state anxiety, and athlete burnout were supported, as were the paths from fear of failure to competitive state anxiety and from competitive state anxiety to athlete burnout. Thus, H1, H2, H3, H4, and H6 were supported. However, the direct path from fear of failure to athlete burnout was not statistically significant in the full structural model; therefore, H5 was not supported. This pattern indicates that fear of failure was not supported as an independent direct correlate of burnout once competitive state anxiety was included, but it remained an important upstream cognitive-appraisal component in the pathway to competitive state anxiety.

### Bootstrapped indirect effects

Bias-corrected bootstrapping with 5,000 resamples was conducted to examine the indirect associations involving fear of failure and competitive state anxiety. Indirect effects were regarded as statistically significant when the 95% confidence interval did not include zero. The results are presented in Table 5. The total effect of socially prescribed perfectionism on athlete burnout was significant (β = 0.561, 95% CI [0.498, 0.620]). After fear of failure and competitive state anxiety were included as mediators, the direct effect remained significant (β = 0.276, 95% CI [0.199, 0.347]), and the total indirect effect was also significant (β = 0.285, 95% CI [0.233, 0.340]). The indirect effect accounted for 50.80% of the total effect.

**Table 5:** Bootstrapping results for direct and indirect effects (*N* = 896).

Path	β	Boot SE	Boot LLCI	Boot ULCI	Ratio (%)
**Direct effect**	0.276^***^	0.037	0.199	0.347	49.20
**Indirect effects**	0.285^***^	0.027	0.233	0.340	50.80
Socially prescribed perfectionism → Fear of failure → Athlete burnout	0.040	0.029	−0.016	0.100	7.13
Socially prescribed perfectionism → Competitive state anxiety → Athlete burnout	0.061^**^	0.025	0.015	0.113	10.87
Socially prescribed perfectionism → Fear of failure → Competitive state anxiety → Athlete burnout	0.184^***^	0.026	0.140	0.240	32.80
**Total effect**	0.561^***^	0.031	0.498	0.620	100

Boot SE, Bootstrap Standard Error; Boot LLCI, Lower Limit of 95% bias-corrected Confidence Interval; Boot ULCI, Upper Limit of 95% bias-corrected Confidence Interval.

Ratio (%) represents the proportion of each effect relative to the total effect.

^*^p < 0.05; ^**^ *p* < 0.01; ^***^ *p* < 0.001.

Regarding specific indirect pathways, the indirect association through fear of failure alone was not significant because the confidence interval included zero; therefore, H7 was not supported. The indirect association through competitive state anxiety alone was significant, supporting H8. The sequential indirect association through fear of failure and competitive state anxiety was also significant, supporting H9. Among the indirect pathways, the sequential pathway accounted for the largest proportion of the total effect.

Taken together, the SEM and bootstrapping results indicated that the proposed model was partially supported and required a more specific interpretation. The association between socially prescribed perfectionism and athlete burnout was not explained by fear of failure alone. Instead, the strongest indirect evidence was found for the sequential pathway in which socially prescribed perfectionism was associated with stronger fear of failure, fear of failure was associated with greater competitive state anxiety, and competitive state anxiety was associated with higher athlete burnout. This pattern is consistent with the proposed cognitive–affective sequence, while also indicating that competitive state anxiety functioned as the more proximal correlate of burnout in the full model.

## Discussion

### Summary of key findings

The present study tested a hypothesized process model linking socially prescribed perfectionism, fear of failure, competitive state anxiety, and athlete burnout in a sample of Chinese competitive athletes. Overall, the findings were consistent with a cognitive–affective indirect-effect pattern in which socially prescribed perfectionism was positively associated with athlete burnout and with both proposed intermediate constructs. Fear of failure was positively associated with competitive state anxiety, and competitive state anxiety was positively associated with athlete burnout. However, the direct association between fear of failure and athlete burnout was not statistically significant once competitive state anxiety was included in the structural model. The bootstrapping results further indicated that the indirect association through fear of failure alone was not significant, whereas the indirect association through competitive state anxiety and the sequential indirect association through fear of failure and competitive state anxiety were significant. Taken together, the findings suggest that the proposed model was partially supported and that the overall pattern is more consistent with a sequential cognitive–affective indirect-effect account than with a simple direct-effect explanation.

### Cognitive–affective interpretation of the supported pathway

A central purpose of this study was to examine whether socially prescribed perfectionism was associated with athlete burnout through a theoretically specified cognitive–affective pathway. In the present model, fear of failure represents the cognitive component because it reflects athletes' appraisal of failure as threatening, shameful, self-devaluing, or socially costly. Competitive state anxiety represents the affective component because it reflects competition-proximal worry, somatic activation, and reduced confidence in evaluative performance situations. Athlete burnout, in turn, represents a more chronic strain outcome characterized by exhaustion, reduced accomplishment, and sport devaluation. This conceptual ordering is consistent with stress-process perspectives in which perceived demands are first appraised, then accompanied by emotional and physiological responses, and only over time become linked to more persistent strain outcomes.

The empirical pattern provided evidence consistent with this cognitive–affective interpretation. Socially prescribed perfectionism was positively associated with fear of failure, suggesting that athletes who perceived stronger external demands for flawlessness also tended to report stronger failure-related threat appraisals. Fear of failure was then positively associated with competitive state anxiety, indicating that these threat appraisals were closely linked to affective responses in competition-related contexts. Competitive state anxiety was positively associated with athlete burnout, suggesting that burnout symptoms were most strongly linked to the proximal affective component of the model. The significant serial indirect effect from socially prescribed perfectionism to athlete burnout through fear of failure and competitive state anxiety therefore supported the proposed cognitive-to-affective sequence.

At the same time, the results did not fully confirm every path proposed in the theoretical model. Compared with the proposed model in Figure 1, the structural results in Figure 2 retained the main sequence from socially prescribed perfectionism to fear of failure, from fear of failure to competitive state anxiety, and from competitive state anxiety to athlete burnout. However, the direct path from fear of failure to athlete burnout was not statistically significant, and the indirect pathway through fear of failure alone was also not significant. Thus, the model should be interpreted as partially supported rather than fully confirmed. Fear of failure appeared to function less as an independent direct correlate of burnout and more as a cognitive appraisal process that became relevant to burnout through its association with competitive state anxiety.

This distinction is important because it clarifies the meaning of the cognitive–affective pathway. The results do not suggest that failure-related threat appraisal alone is sufficient to explain burnout-related symptoms. Instead, the findings indicate that cognitive appraisal and affective strain may need to be considered together. Failure-related appraisals may heighten the perceived stakes of competition, but their association with burnout appears to be stronger when they are accompanied by competition-related anxiety. Because the study used a cross-sectional design, these findings should be interpreted as evidence of an association pattern consistent with the proposed cognitive–affective sequence, not as proof of temporal or causal ordering.

### Socially prescribed perfectionism as a distal correlate of athlete burnout

The positive association between socially prescribed perfectionism and athlete burnout is consistent with conceptualizations of burnout as a stress-related outcome in evaluative achievement settings (Flett et al., 1994). Socially prescribed perfectionism reflects the perception that others expect flawless performance and attach interpersonal or status-related consequences to imperfection. In sport, such perceptions may be especially salient because performance is publicly observable, feedback can be immediate, and selection outcomes are consequential. Within this context, the present findings are compatible with the view that externally imposed perfectionistic expectations are linked to burnout-related strain.

This finding is also consistent with broader evidence showing that perfectionism is associated with burnout in athletes, particularly when perfectionism involves evaluative concerns and externally imposed standards (Yang et al., 2023). The current results extend this line of work by focusing specifically on socially prescribed perfectionism as a perceived interpersonal demand. Rather than treating perfectionism only as an individual tendency toward high standards, the present study emphasizes the social-evaluative quality of this dimension: athletes may experience performance as something that must satisfy coaches, teammates, family members, or organizations. In such conditions, mistakes may carry psychological costs beyond poor performance itself.

The broader pattern of associations also suggests that socially prescribed perfectionism may be relevant not only as a direct correlate of burnout symptoms but also as a background evaluative orientation associated with how athletes interpret competitive demands. In the present sample, socially prescribed perfectionism was linked to both fear of failure and competitive state anxiety, which is consistent with the possibility that athletes who perceive stronger external performance demands are also more likely to report stronger failure-related threat appraisals and greater competition-related tension. Thus, rather than viewing socially prescribed perfectionism solely as an isolated personality correlate, the current findings are more compatible with a broader stress-appraisal perspective in which this form of perfectionism is associated with both cognitive and affective aspects of athletes' responses to evaluative sport contexts.

### Fear of failure as a cognitive amplifier rather than a direct correlate of burnout

The present findings offer a more differentiated interpretation of fear of failure in relation to athlete burnout (Birr et al., 2024). Fear of failure was strongly associated with competitive state anxiety, which is in line with the view that failure-related threat appraisals are closely tied to pre-competition worry and somatic tension (Lee and Kang, 2024). When athletes assign substantial personal and interpersonal costs to mistakes, competition may be appraised less as a challenge and more as a threat, thereby increasing vigilance, anticipatory concern, and self-monitoring. This interpretation is broadly consistent with appraisal-based models in which threat evaluations are closely linked to anxiety responses in demanding performance environments (Harris et al., 2023).

The broader fear-of-failure literature also supports the interpretation of fear of failure as a cognitive appraisal process that can influence motivation, avoidance, and affective experiences in sport and exercise contexts (Taylor et al., 2023). In the present model, fear of failure was not simply another negative emotional state. Rather, it represented the athlete's interpretation of what failure would mean for self-worth, social regard, and future standing. This helps explain why fear of failure was strongly linked to competitive state anxiety: when athletes appraise possible failure as personally or socially damaging, the upcoming competition may become an anxiety-provoking event.

However, fear of failure did not show a statistically significant direct association with athlete burnout in the full structural model. This pattern suggests that fear of failure may not function as a direct burnout correlate once more proximal affective responses are taken into account. Instead, the findings are more compatible with the idea that fear of failure acts as a cognitive amplifier: it may intensify the perceived stakes of competition and thereby become linked to burnout primarily through heightened competition-related anxiety. This interpretation also helps explain why fear of failure is often implicated in sport-related distress while not always showing a robust direct association with more chronic outcomes when proximal emotional states are modeled simultaneously (Kensbock and Stöckmann, 2025).

### Competitive state anxiety as a proximal correlate of athlete burnout

Competitive state anxiety emerged as the construct most proximally associated with athlete burnout in the structural model (Yang et al., 2024). This finding is compatible with the notion that repeated episodes of competition-related worry and physiological tension may be linked to broader patterns of exhaustion, reduced accomplishment, and sport devaluation when they recur across training and competition cycles (Wu et al., 2022). In other words, the results are consistent with the possibility that athletes' day-to-day or competition-to-competition affective experiences may matter greatly for whether more chronic burnout symptoms are reported.

The role of competitive state anxiety is also consistent with evidence that competitive anxiety varies as a function of athlete characteristics and sport-context factors, indicating that anxiety responses are sensitive to evaluative and performance-related conditions (Rocha and Osório, 2018). In highly evaluative sport environments, anxiety may become a recurring affective response to competitions, selection events, and performance feedback. When such anxiety is repeated, it may require continuous self-regulation of worry, arousal, and attentional focus. This self-regulatory demand can increase perceived effort costs and may contribute to the exhaustion and motivational erosion that characterize burnout.

The significant indirect association through competitive state anxiety, together with the stronger sequential indirect association involving fear of failure and competitive state anxiety, further highlights the importance of competition-related affective strain in understanding burnout in evaluative sport environments (Kwon and Cho, 2020; Yang et al., 2024). This interpretation is consistent with stress-burnout evidence showing that athlete stress is positively associated with burnout symptoms (Lin et al., 2022). At the same time, because the study was cross-sectional, this should not be interpreted as demonstrating that anxiety causes burnout. A more cautious interpretation is that burnout symptoms were most strongly linked to the anxiety component of the model, and that the overall pattern is compatible with stress-process accounts in which proximal affective strain occupies an important position between evaluative pressure and burnout-related outcomes.

### Serial indirect-effect pattern and hypothesis interpretation

The combined SEM and bootstrapping results were consistent with a sequential indirect-effect pattern in which socially prescribed perfectionism was associated with fear of failure, fear of failure was associated with competitive state anxiety, and competitive state anxiety was associated with athlete burnout (Kwon and Cho, 2020). Importantly, the largest indirect association was the sequential one involving both mediators, whereas the indirect association through fear of failure alone was not significant. This pattern refines the interpretation of the hypotheses beyond a simple direct-effects account.

More specifically, the direct-association hypotheses were largely supported: socially prescribed perfectionism was positively associated with athlete burnout, fear of failure, and competitive state anxiety; fear of failure was positively associated with competitive state anxiety; and competitive state anxiety was positively associated with athlete burnout. The only unsupported direct hypothesis was the path from fear of failure to athlete burnout. In substantive terms, this pattern suggests that fear of failure may be less relevant as an independent correlate of burnout than as a cognitive appraisal process associated with stronger anxiety responses (Kensbock and Stöckmann, 2025; Minichiello et al., 2024).

The indirect-effect hypotheses also showed a differentiated structure. The non-significant indirect association through fear of failure alone indicates that failure-related appraisals may not, by themselves, be sufficient to account for the link between socially prescribed perfectionism and burnout. By contrast, the significant indirect association through competitive state anxiety and the significant sequential indirect association suggest that burnout is more closely linked to the combination of evaluative threat appraisal and competition-related affective strain than to fear of failure in isolation (Taleshmekaeil et al., 2025). This interpretation is also in line with process-oriented perspectives in which antecedent appraisals matter most when they are accompanied by downstream affective activation and self-regulatory burden (Cashman et al., 2024; Rumbold et al., 2023).

### Theoretical implications

Several theoretical implications follow from the present findings. First, the study extends perfectionism research in sport by showing that the association between socially prescribed perfectionism and athlete burnout is better understood within a broader stress-process framework than as a purely bivariate relation (Kwon and Cho, 2020). The present results are consistent with the view that socially prescribed perfectionism is linked not only to burnout symptoms themselves but also to failure-related appraisals and competition-related affective strain. This is important because it positions socially prescribed perfectionism as a distal social-evaluative pressure rather than merely as a general maladaptive personality correlate.

Second, the findings help differentiate the roles of fear of failure and competitive state anxiety. In the present model, fear of failure was more closely aligned with the cognitive appraisal component of the process, whereas competitive state anxiety was more closely aligned with the proximal affective component. This distinction is theoretically useful because it suggests that not all negative psychological responses to evaluative pressure operate at the same level. Appraisal-oriented constructs may help explain how athletes interpret pressure, whereas proximal emotional states may be more closely associated with whether that pressure is accompanied by burnout-related symptoms (Kang and Gong, 2024).

Third, the serial indirect-effect pattern contributes to athlete burnout research by highlighting a plausible cognitive-to-affective sequence linking perceived external pressure and burnout-related outcomes (Olsson et al., 2022). Rather than implying that externally imposed perfectionistic expectations directly translate into burnout, the findings are more compatible with a layered interpretation: social expectations may be associated with stronger failure-related threat appraisals, which in turn may be associated with greater competition-related anxiety, and this overall pattern may be linked to burnout symptoms. This process-oriented framing helps connect interpersonal pressure constructs with burnout research and suggests that models focusing only on direct paths may miss important transitions between pressure, appraisal, and emotional strain (Xu et al., 2024).

### Practical implications

The present findings also have practical implications for applied sport settings. At the environmental level, they underscore the importance of how evaluative climates are communicated and managed by coaches and sport organizations (Gómez-López et al., 2025). If athletes perceive approval and evaluation as contingent on flawless performance, the conditions may become more favorable for fear of failure and competition-related anxiety. Accordingly, feedback practices that normalize mistakes as part of learning, reduce public blame, and emphasize controllable process goals may help reduce the perceived interpersonal costs of imperfection (Abós et al., 2021; Porter et al., 2024). Similarly, transparent and development-oriented selection procedures may reduce ambiguity and lessen the extent to which athletes interpret evaluation as conditional acceptance.

At the individual level, the observed pattern suggests that intervention efforts may need to target both appraisal processes and competition-related anxiety rather than focusing exclusively on one or the other. Because fear of failure was not directly associated with burnout in the full model, interventions aimed only at changing failure-related beliefs may not fully address burnout-related risk (Alecu and Onea, 2025). Cognitive work on reframing the consequences of failure may still be important, but the findings suggest that such work may be most effective when combined with strategies aimed at reducing maladaptive pre-competition activation, such as arousal regulation, attentional control training, pre-competition routines, and coping planning (Sharpe et al., 2024). In practical screening contexts, socially prescribed perfectionism may be useful as a more distal risk marker, whereas competitive state anxiety may serve as a more proximal warning sign.

### Limitations and future directions

Several limitations should be acknowledged. First, the study used a cross-sectional design, which does not permit causal inference or firm conclusions about temporal ordering. Although the hypothesized sequence was theory-based and the observed pattern of indirect effects was consistent with that sequence, the data cannot establish that socially prescribed perfectionism preceded fear of failure, that fear of failure preceded competitive state anxiety, or that these processes unfolded over time before burnout symptoms emerged. Longitudinal, diary, and prospective panel designs would be especially valuable for testing whether these constructs show the temporal ordering implied by the current model.

Second, all focal constructs were assessed through self-report measures administered in a single online survey. Although procedural remedies and multiple statistical diagnostics suggested that common method bias was unlikely to fully account for the findings, shared method variance remains possible. Future studies could strengthen inference by incorporating multiple sources of information, such as coach ratings, observer-based climate assessments, competition records, or physiological indicators relevant to pre-competition arousal and recovery.

Third, the sample was heterogeneous with respect to sport type, competitive level, age, and training background, and recruitment was conducted through coaches, team managers, and training units. The present findings therefore represent average associations across the overall sample and should not be assumed to be identical across athlete subgroups. Although this sampling strategy improved access to a broad competitive-athlete population, it also means that responses may not have been fully independent. Athletes nested within the same team, coach, or training system may share experiences that were not modeled explicitly in the present analyses. Future work should therefore consider multilevel or cluster-adjusted approaches and, where possible, collect identifiers that allow team-, coach-, or unit-level nesting to be modeled directly.

Fourth, background characteristics such as gender, sport type, competition level, and years of training were not included as routine covariates in the primary SEM analyses because the model was designed to test a theory-driven psychological process rather than a covariate-adjusted prediction model. Even so, it remains possible that the observed associations differ across athlete subgroups. Future research should examine whether the present pattern is invariant across gender, individual vs. team sports, and competitive level, for example through multigroup SEM or formal measurement-invariance testing.

Fifth, all measures were administered using a harmonized five-point response format and refined Chinese-language versions. This strategy was adopted to reduce response burden and improve comparability across instruments in a single online survey, but it also means that the present administration format differed from the original response anchors of several source instruments. Although the psychometric results in the present sample were acceptable, future studies should further test the equivalence of these adapted response formats and compare them with validated Chinese versions using their original response structures.

Finally, competitive state anxiety was modeled as a broader latent construct defined by cognitive anxiety, somatic anxiety, and reverse-scored self-confidence. This operationalization was theoretically defensible for the present model, but it may also obscure potentially different roles of the three CSAI-2R components. Future research should examine whether cognitive anxiety, somatic anxiety, and self-confidence show distinct associations with burnout and whether the present results are robust when these components are modeled separately.

## Conclusion

This study examined whether the association between socially prescribed perfectionism and athlete burnout in Chinese competitive athletes was consistent with a cognitive–affective indirect-effect pattern involving fear of failure and competitive state anxiety. The findings showed that socially prescribed perfectionism was positively associated with athlete burnout, fear of failure, and competitive state anxiety; fear of failure was positively associated with competitive state anxiety; and competitive state anxiety was positively associated with athlete burnout, whereas fear of failure was not directly associated with athlete burnout in the full structural model. Bootstrapping further indicated that the indirect association through fear of failure alone was not significant, while the indirect association through competitive state anxiety and the sequential indirect association through fear of failure and competitive state anxiety were significant. Overall, the pattern of findings was most consistent with a serial indirect-effect model in which externally imposed perfectionistic expectations were linked to burnout symptoms through failure-related threat appraisal and competition-related anxiety. In this model, fear of failure represents the cognitive appraisal component, whereas competitive state anxiety represents the affective competition-proximal response component. Given the cross-sectional design, these results should be interpreted as consistent with, rather than definitive evidence for, a cognitive–affective process linking socially prescribed perfectionism to athlete burnout.

## Data Availability

The datasets presented in this study can be found in online repositories. The names of the repository/repositories and accession number(s) can be found below: The dataset supporting this study is publicly available in the Zenodo repository at https://doi.org/10.5281/zenodo.19518683. This DOI is the Zenodo concept DOI, which represents all versions of the record and resolves to the latest version.
